# Does toxicity of aromatic pollutants increase under remote atmospheric conditions?

**DOI:** 10.1038/srep08859

**Published:** 2015-03-09

**Authors:** Ana Kroflič, Miha Grilc, Irena Grgić

**Affiliations:** 1 Analytical Chemistry Laboratory, National Institute of Chemistry, Hajdrihova 19, SI-1001 Ljubljana, Slovenia; 2 Laboratory of Catalysis and Chemical Reaction Engineering, National Institute of Chemistry, Hajdrihova 19, SI-1001 Ljubljana, Slovenia

## Abstract

Aromatic compounds contribute significantly to the budget of atmospheric pollutants and represent considerable hazard to living organisms. However, they are only rarely included into atmospheric models which deviate substantially from field measurements. A powerful experimental-simulation tool for the assessment of the impact of low- and semi-volatile aromatic pollutants on the environment due to their atmospheric aqueous phase aging has been developed and introduced for the first time. The case study herein reveals that remote biotopes might be the most damaged by wet urban guaiacol-containing biomass burning aerosols. It is shown that only after the primary pollutant guaiacol has been consumed, its probably most toxic nitroaromatic product is largely formed. Revising the recent understanding of atmospheric aqueous phase chemistry, which is mostly concerned with the radical nitration mechanisms, the observed phenomenon is mainly attributed to the electrophilic nitrogen-containing reactive species. Here, their intriguing role is closely inspected and discussed from the ecological perspective.

Placing biomass in a wider context, it can be considered the most important energy source. As it is of a natural origin, it does not contribute any net carbon dioxide to the atmosphere, if the collection and transport are not regarded. Historically, biomass was extensively used for direct heat production via wood combustion and nowadays it is very attractive because of its substantial potential to meet the challenges of green energy. Despite the mounting controversy over biodiesel nitrogen oxide emissions and other related ecological issues^1^,^2^, it would not be surprising if biomass became the leading renewable material for sustainable production of biofuels and chemicals in the foreseeable future.

Although anthropogenic biomass burning (BB) in principle avoids excess greenhouse-gas emissions, it significantly alters the atmospheric absorption of solar irradiation, even in remote regions^3^,^4^, and thus influences the Earth's climate directly. Brown carbon (BrC), which is considered a light-absorbing organic matter (OM), together with black carbon represent major absorbing components in atmospheric aerosols. Both are closely related to biomass combustion, but the wavelength dependent extinction of the former makes the evaluation of its impact on the overall atmosphere's aerosol absorption very intricate^5^. Wood smoke particulate mass (PM) was found to contain up to 40% phenol derivatives, which are common light-absorbing OM^6^. Moreover, anthropogenic BB was recently estimated to account for 50% of the total anthropogenic atmospheric OM^7^, whilst only BB plume aging could contribute up to 5% to the total atmospheric organic aerosol (OA) budget^8^. At this point it should be emphasized, that atmospheric OM not only alters the Earth's climate by its impact on the troposphere's scattering, but indirectly also by affecting cloud properties through governing the particle hygroscopicity and the ability of particles to act as cloud condensation nuclei.

Besides being an important source of tropospheric OA, lignocellulosic biomass also contributes substantially to atmospheric BrC. During the pyrolysis of the aromatic polymer lignin, semi-volatile organic compounds (SVOC), such as guaiacol (GUA), are emitted into the troposphere. These compounds can remain in the gaseous phase, but also partition into the atmospheric aqueous phase. They are further transformed by atmospheric reactive species and can yield new airborne pollutants. Their aged oxidation products are mostly less volatile, more water soluble, able to absorb light and capable of forming secondary organic aerosols (SOA)^9^,^10^,^11^,^12^,^13^,^14^,^15^. Especially the nitro-aromatic derivatives are regarded as potent toxic, mutagenic, or carcinogenic compounds and represent considerable danger to human health and other living organisms^16^,^17^,^18^. They are often coloured and seem promising candidates for forming SOA^19^,^20^,^21^. Nitrogen-containing organic compounds have been detected in BB plume^22^ and nitrated derivatives of GUA have been recently determined in ambient aerosol samples^23^.

Based on Henry's law constants, concentrations of organic pollutants in atmospheric waters are often predicted to be very low. Nevertheless, it is believed that their aqueous phase transformations play a more important role in the troposphere than deduced from the theory and recent knowledge^24^,^25^,^26^. If aqueous phase processes were properly addressed by atmospheric models, which deviate substantially from field measurements, the estimation of the organic O/C ratio and the BrC and SOA abundance would be significantly altered^27^,^28^. Atmospherically relevant aqueous phase chemistry has thus been closely inspected for the past decade. Extensive studies have revealed the pronounced effect of aqueous phase transformations on aging of SVOCs, and their subsequent partitioning between atmospheric gas and aqueous phase, in conditions of cloud droplets, fog, and wet aerosol particles^29^.

An intriguing fate of the BB pollutant GUA in the atmospheric aqueous phase is emphasized in this manuscript. Investigation of its aging in originally urban deliquescent aerosols is based on a long-term kinetic study of GUA nitration in acidic H_2_SO_4_ solution (pH 4.5), typical for the atmospheric waters. Addition of sodium nitrite (NaNO_2_) and hydrogen peroxide (H_2_O_2_) into the reaction mixture allowed us to deduce the reactivity of GUA and its primary nitrated products (4-nitroguaiacol (4NG) and 6-nitroguaiacol (6NG)) with different nitrogen-containing reactive species (NRS). To address the diurnal cycle, experiments were performed in the dark and under simulated sunlight. Using the kinetic parameters obtained from the experiments, simulation studies were performed to compare transformations of GUA under urban and remote atmospheric conditions.

## Results

### Experimental and modelling

Based on the extensive experimental study of GUA nitration in aqueous solution under different atmospherically relevant conditions a reaction mechanism was proposed (Supplementary Fig. 1), model function derived and fitted simultaneously to all experimental data, yielding a good correlation (R^2^ = 0.9983). Such approach allowed us to determine the kinetic constants for GUA, 4NG, and 6NG reacting with nitrite (NO_2_^·^) and nitroso (NO^·^) radicals as well as nitronium (NO_2_^+^) and nitrosonium (NO^+^) ions with a great deal of confidence (Supplementary Table 1). The concentration profiles of the NRSs in the reaction mixture during the experiments were also retrieved from the theoretical model (Supplementary Figs. 4a and 5a) and are crucial for the following discussion. In contrast to most other studies of aqueous phase transformations of aromatic pollutants, GUA nitration cannot be simply explained by the steady-state concentrations of radical species in solution. Our experimental-modelling approach revealed that, beside the concentration-dependent profiles of nitrogen-containing radicals, changing concentrations of electrophilic nitrating agent are also required to properly describe the experimental data. Optimized kinetic rate constants are gathered in Supplementary Table 1 and results of the fitting procedure are shown in Supplementary Figs. 2 and 3, next to the experimental data obtained in the dark and under simulated sunlight conditions, respectively. In addition, results of the fitting procedure are shown in comparison to the modelled concentration profiles according to the radical and electrophilic mechanism only (Supplementary Figs. 4b and 5b). Predicted lifetimes of GUA are also reported for each reaction mechanism.

### Simulations

**Figure 1:**
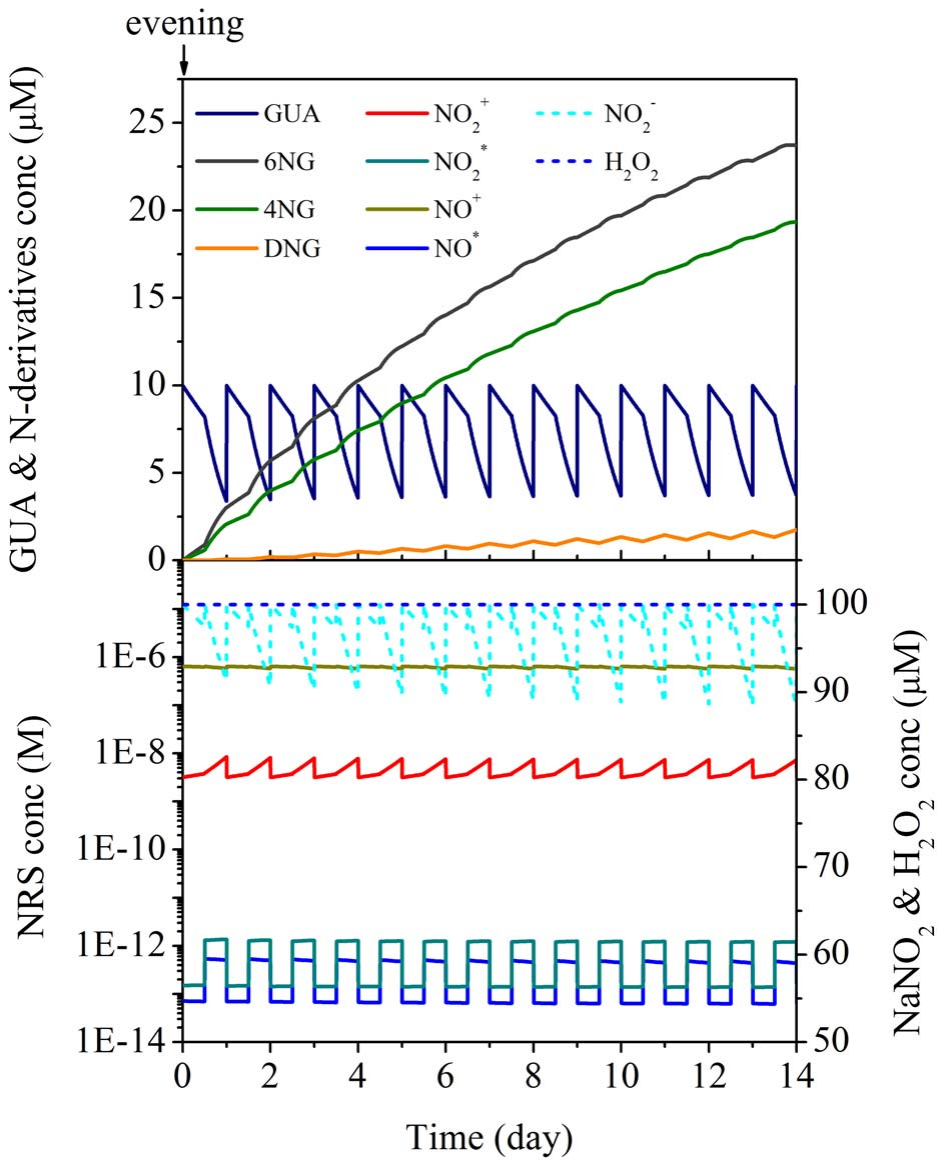
Urban case simulation study. Consecutive evening BB events in the urban area with anthropogenic NO_x_ emissions during the daytime. Concentration of GUA (solid dark blue line) in the simulation study was adjusted to 0.01 mM every 24 hours (in the simulated evening) and that of NaNO_2_ (dashed cyan line) was held constant at 0.1 mM through the daytime (for every second 12 hours). The concentration of H_2_O_2_ (dashed blue line) was held constant throughout the simulation study at 0.1 mM. Temperature and pH were assumed to be 25°C and 4.5, respectively. Other symbols used: 4NG (solid green line), 6NG (solid dark grey line), DNG (solid orange line), NO_2_^·^ (solid dark cyan line), NO^·^ (solid blue line), NO_2_^+^ (solid red line), and NO^+^ (solid dark yellow line).

**Figure 2:**
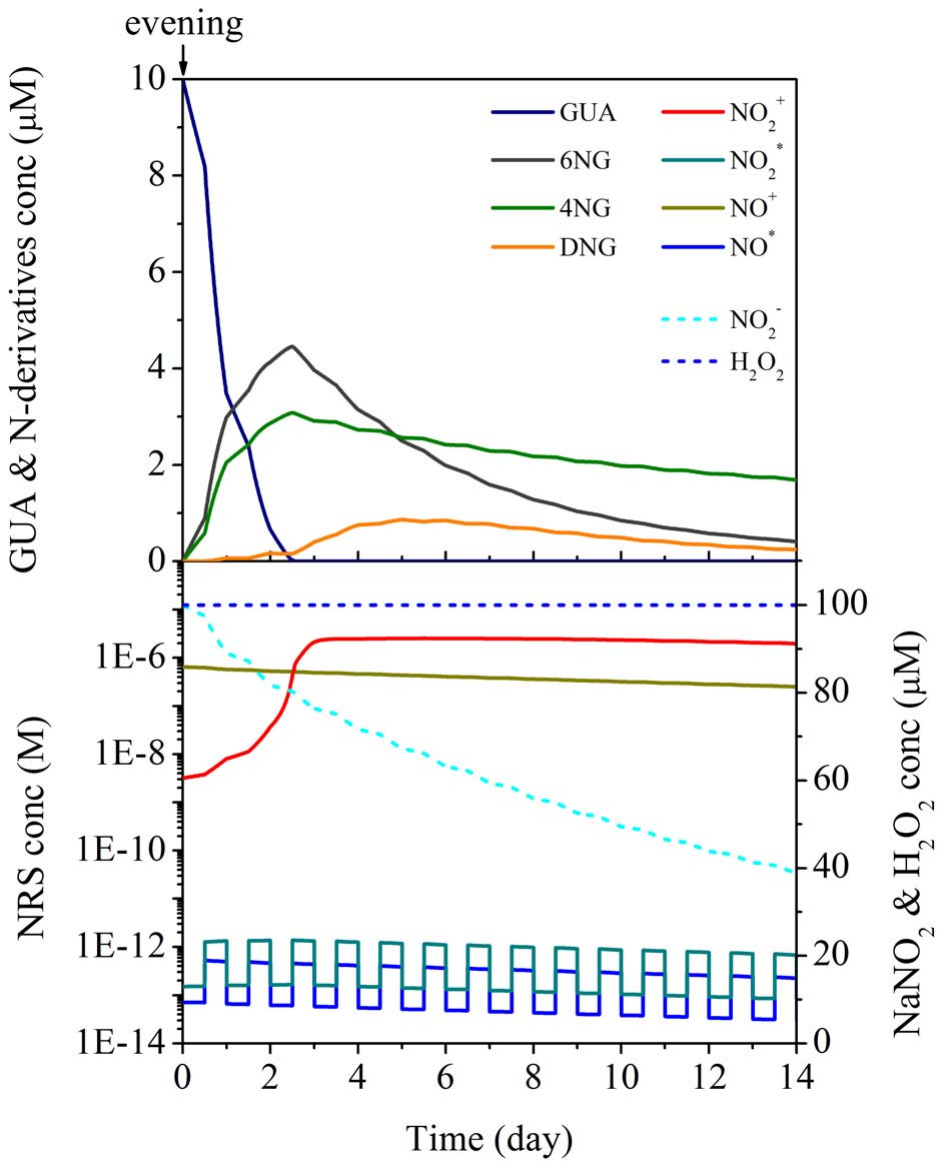
Remote case simulation study. Single evening BB event in the urban area with anthropogenic NO_x_ emissions during the daytime, followed by migration of the formed deliquescent OA toward remote regions with no new emissions of either GUA or NO_x_. Initial concentrations of GUA (solid dark blue line) and NaNO_2_ (dashed cyan line) used in the simulation study were 0.01 mM and 0.1 mM, respectively. Concentration of H_2_O_2_ (dashed blue line) was held constant throughout the simulation study at 0.1 mM. Temperature and pH were assumed to be 25°C and 4.5, respectively. Other symbols used: 4NG (solid green line), 6NG (solid dark grey line), DNG (solid orange line), NO_2_^·^ (solid dark cyan line), NO^·^ (solid blue line), NO_2_^+^ (solid red line), and NO^+^ (solid dark yellow line).

The proposed theoretical model of GUA aging in aqueous solution (Supplementary Fig. 1) was used to simulate its fate in deliquescent aerosols by mimicking the diurnal cycle and considering two distinct scenarios: i) consecutive evening BB events in an urban area with anthropogenic NO_x_ emissions during the daytime (urban case) and ii) single evening BB event in an urban area with anthropogenic NO_x_ emissions during the daytime, followed by migration of the formed deliquescent OA toward remote regions with no new emissions of either GUA or NO_x_ (remote case). In both simulations starting with an evening BB event, kinetic constants valid at the applied experimental conditions were used (25°C, pH 4.5). For other simulation parameters (concentrations of GUA, NaNO_2_, and H_2_O_2_) please refer to Methods section. Results of the simulation study are shown in Figs. 1 and 2 for urban and remote atmospheric conditions, respectively.

## Discussion

In the presented experimental-simulation study, a special attention was paid to the yellow-coloured secondary product 4,6-dinitroguaiacol (DNG) which could be, similarly to 2,4-dinitrophenol, particularly hazardous to plants and aquatic organisms^30^. Unfortunately, the harmful potential of DNG in comparison to the mono-nitro derivatives of GUA has not been evaluated yet; however the QSAR study on nitroaromatics toxicity suggests that it most likely increases with nitro-substitution of the aromatic ring^31^. Besides the probable exertion of damage on biotopes by the deposition of toxic aerosol particles, DNG also affects sunlight scattering and the local climate as a constituent of BrC.

It is evident from the long-term experimental study presented in Supplementary Fig. 3 (upper two diagrams) that DNG production is substantially accelerated after GUA has been completely consumed. This surprising fact was the strongest motivator for inclusion of the electrophilic species into the proposed reaction model, since the radical mechanism does not allow accounting for such drastic changes. Whereas free radicals typically react with high absolute rate constants, the reactivity of electrophiles toward aromatic compounds differs substantially^32^. In our case, GUA's electron-donating –OH and –OCH_3_ groups highly activate the aromatic ring, but the electron-withdrawing –NO_2_ substituent in nitroguaiacol significantly decreases its reactivity to electrophilic attack. The importance of electrophiles in environmental chemistry has already been speculated^33^,^34^; however, to the best of our knowledge, this is the first time that they are explicitly taken into consideration when addressing the transformations of organic compounds in atmospheric waters. In the next paragraph, the very important consequences, arising from the nature of electrophilic nitrating agent, are outlined.

The apparent influence of the precursor aromatic on the nitration of its primary products absolutely calls for opening the discussion on the correlation between BB source proximity and the exerted hazards of aged OA on the environment. To address this very important issue, the simulation studies represented in Figs. 1 (the urban case) and 2 (the remote case) were compared. The results therein show that according to the remote scenario the predicted concentration of DNG in 4.5 days old wet aerosol is 1.9 times higher than in the urban case OA. Furthermore, the cumulative amounts of GUA emitted and DNG produced in each scenario reveal that in the remote case the transformation of GUA into DNG is highly prevalent (up to 9.3 times more DNG is formed from the same amount of GUA than in the urban case in the same OA aging time). Even though temperature and pH alter the kinetic constants considerably (based on the preliminary studies, opposing effects of temperature and pH on the reaction kinetics are expected in more acidic aerosols at lower temperature), it can be concluded that harmful effects of GUA, aged in atmospheric wet aerosol particles and initially accompanied by anthropogenic NO_x_ emissions, would be intensified in remote regions, if it was correctly assumed that DNG is much more toxic than 4NG and 6NG. Furthermore, this finding can be generalised to other aromatic pollutants, as the formation of dinitroaromatics in atmospheric aerosols is expected to accelerate after the highly reactive methoxyphenols have been consumed. Therefore, in the absence of the primary pollutants the production of di-nitro derivatives is substantially enhanced, which suggests accumulation of the presumably most toxic products in the regions downwind of the BB source and, most importantly, emphasizes a special threat for remote biotopes, i.e. rain forests and oceans.

**Figure 3:**
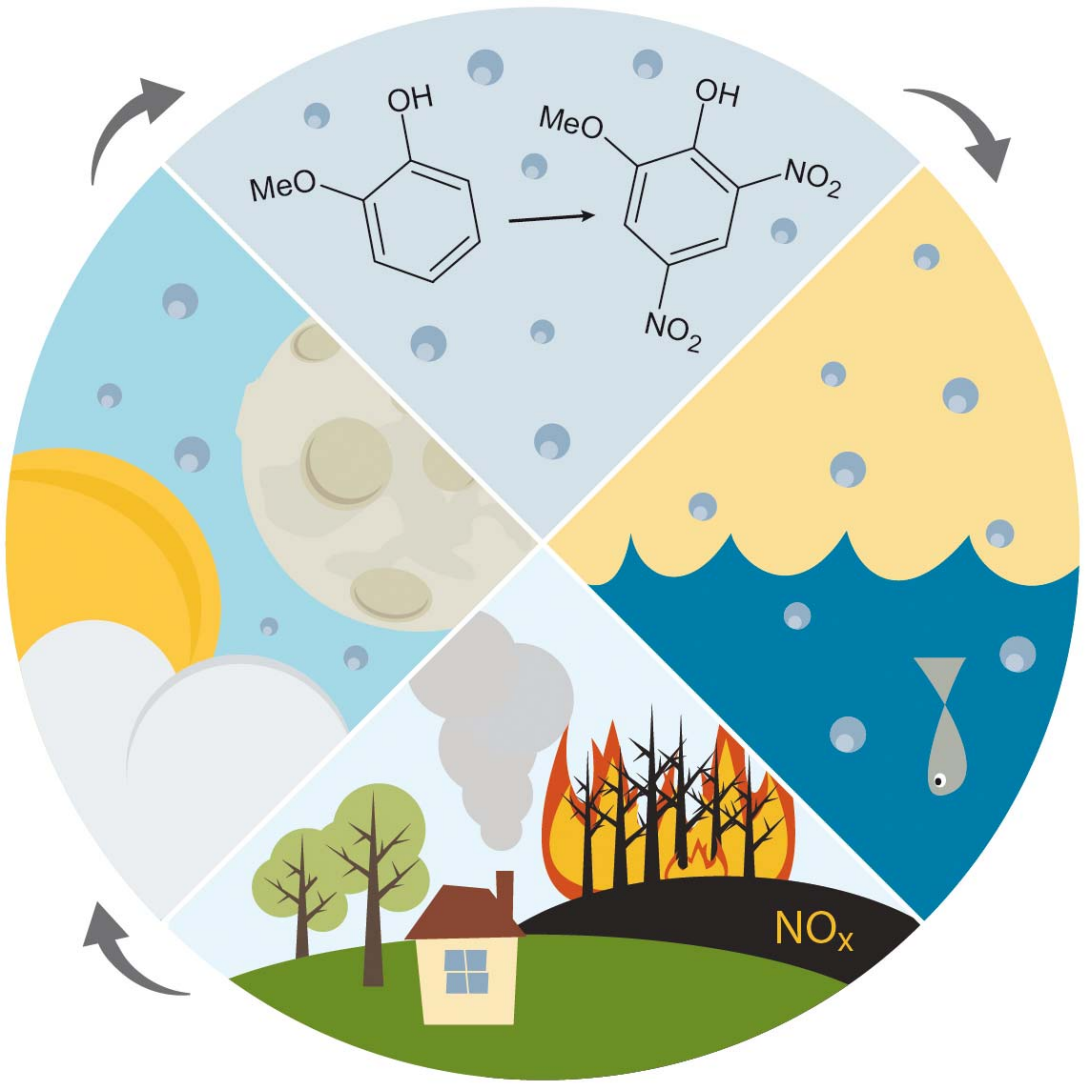
From emissions to remote regions. Scheme represents an environmental cycle of a biomass burning pollutant guaiacol and emphasizes its particularly harmful effect on remote biotopes.

The reported novel approach to study the reactivity of SVOCs in the atmospheric aqueous phase is shown as a powerful and very promising tool for forecasting the pollutants' fate in the tropospheric waters and will be further developed. The authors expect that with taking into account changing environmental conditions and movements of air masses within the troposphere, the influence of distinct pollutants on the specific remote biotopes will be adequately addressed in the future. The present work identifies electrophiles as remarkable reactive species in acidic atmospheric waters, contributing essentially to better understanding of the wet OA aging and emphasising the ecological perspective. An environmental cycle of GUA is illustrated in Fig. 3.

## Methods

### Reagents

Acetonitrile (Chromasolv gradient grade, for HPLC, ≥ 99.9%), tetrahydrofuran (Chromasolv Plus, for HPLC, ≥ 99.9%, inhibitor-free), ammonium formate (Puriss *p.a.*, eluent additive for LC/MS), formic acid (Puriss *p.a.*, eluent additive for LC/MS) and high purity water (18.2 MΩ cm), supplied by a Milli-Q water purification system, were used for mobile phase preparation. Sulfuric acid 98% (analysis grade), sodium nitrite (ACS reagent, ≥ 97.0%), hydrogen peroxide 30% (Perhydrol, for analysis), and vitamin C (ascorbic acid, puriss *p.a.*, ≥ 99.0%) were used for reaction mixture preparation and quenching. The following standard substances were used also as reactants: guaiacol (GUA), 4-nitroguaiacol (4NG), 2-methoxy-6-nitrophenol (6-nitroguaiacol, 6NG), and 4,6-dinitroguaiacol (DNG, produced by Kitanovski *et al.*^35^). Purity of all of the standards was ≥ 97% and they were used without further purification. Griess reagent (modified) was used for spectrophotometric determination of nitrite.

### Experimental

Nitration of GUA, 4NG, and 6NG in acidic H_2_SO_4_ solution (pH 4.5) was investigated in the dark and under simulated sunlight conditions with use of the following experimental setup: a solar simulator LOT-QuantumDesign Europe, equipped with an ozone free xenon short arc lamp operated at 250 W, while the reaction mixture in a DURAN® flask was held at the specified distance, thermostated to 25°C in a thermostated bath, and thoroughly mixed by rotation. For dark experiments, an amber DURAN® flask was used without the solar simulator being turned on. Nitration reaction was started each time with addition of NaNO_2_ into the acidic reaction mixture containing GUA, 4NG, or 6NG with or without H_2_O_2_. Initial concentrations of reactants in the reaction mixture were 0.1 mM GUA, 0.02 mM 4NG, or 0.023 mM 6NG and 1 mM NaNO_2_. When H_2_O_2_ was added to the reaction mixture, its initial concentration was adjusted to 1 mM. A 0.5 mL aliquot was taken from the reaction mixture at each scheduled time and quenched by 1% ascorbic acid (vitamin C) as the most suitable reaction quencher^35^. The concentrations of GUA, 6NG, 4NG, and DNG were determined immediately using an Agilent 1100 Series HPLC system equipped with a UV/Vis diode-array detector (DAD). The separation was performed on an Atlantis T3 column (3.0 × 150 mm, 3 μm particle size, Waters) connected to an Atlantis T3 guard column (3.9 × 20 mm, 3 μm particle size, Waters) with an isocratic mobile phase acetonitrile/tetrahydrofuran/water (30/4/66, v/v/v) containing 7.5 mM formic acid/ammonium formate buffer, pH 3, at a flow rate of 0.5 mL min^−1^. The injection volume and column temperature were 100 μL and 30°C, respectively.

Based on duplicate injections, the precision of the measurement was within ± 5%. The long-term concentration profiles are composed of two distinct experiments performed under the same experimental conditions. The data in 0–12 and 24–36 h time intervals correspond to the same experiment, whereas another experiment was performed to obtain the data points in 12–24 and 36–44 h time intervals. In addition, the third reaction was performed under the same conditions to confirm the repeatability of the experiments. All data points obtained under the same experimental conditions are gathered on one diagram and show good matching.

Nitrite concentration was measured spectrophotometrically by use of Griess reagent for nitrite mass balance control.

### Modelling

Optimized kinetic rate constants correspond to the minimum of the objective function, i.e. the sum of squares of the difference between the experimental value and the value determined by solving the set of differential molar balances, derived according to the reaction scheme in Supplementary Fig. 1. For each set of experimental data the experimentally determined initial concentration of GUA, 6NG, or 4NG is taken into account in the fitting procedure. Nelder-Mead method was initially applied for the approximate optimisation of kinetic rate constants, followed by Levenberg-Marquardt optimisation method for the final parameters' optimisation and Jacobian matrix computation, required for the subsequent determination of confidence intervals.

### Simulations

For predictive simulation models the best-fit kinetic parameters obtained from the fitting procedure and valid at the experimental conditions applied (25°C and pH 4.5) were used. Based on the literature data survey, ten times lower reactant concentrations as described in Experimental section were assumed in the simulation studies to better account for the atmospheric conditions^30^,^34^,^36^,^37^. Initial amounts of GUA and NaNO_2_ in the simulated wet aerosols were taken to be 0.01 mM and 0.1 mM, respectively. Concentration of H_2_O_2_ was held constant in both cases (0.1 mM). For more detailed description of the simulation studies please refer to Fig. captions.

## Author Contributions

A.K. performed the laboratory experiments, analysed the experimental results, and wrote the article. M.G. performed the fitting procedure and simulation studies. I.G. significantly participated in discussions concerning the work presented. All authors reviewed the manuscript.

## Supplementary Material

Supplementary Information Does toxicity of aromatic pollutants increase under remote atmospheric conditions?
