# Supplementary material for: Does toxicity of aromatic pollutants increase under remote atmospheric conditions?
Source: Sci Rep. 2015 Mar 9;5:8859. doi: 10.1038/srep08859 (PMC4352892; doi:10.1038/srep08859)
Supplement: Supplementary Information — Does toxicity of aromatic pollutants increase under remote atmospheric conditions? [file srep08859-s1.pdf]

Supplementary Information for

Does toxicity of aromatic pollutants increase under remote atmospheric conditions?

Ana Kroflič[1,*], Miha Grilc[2] & Irena Grgić[1]

[1] Analytical Chemistry Laboratory, National Institute of Chemistry, Hajdrihova 19, SI-1001 Ljubljana, Slovenia.

[2] Laboratory of Catalysis and Chemical Reaction Engineering, National Institute of Chemistry, Hajdrihova 19, SI-1001 Ljubljana, Slovenia.

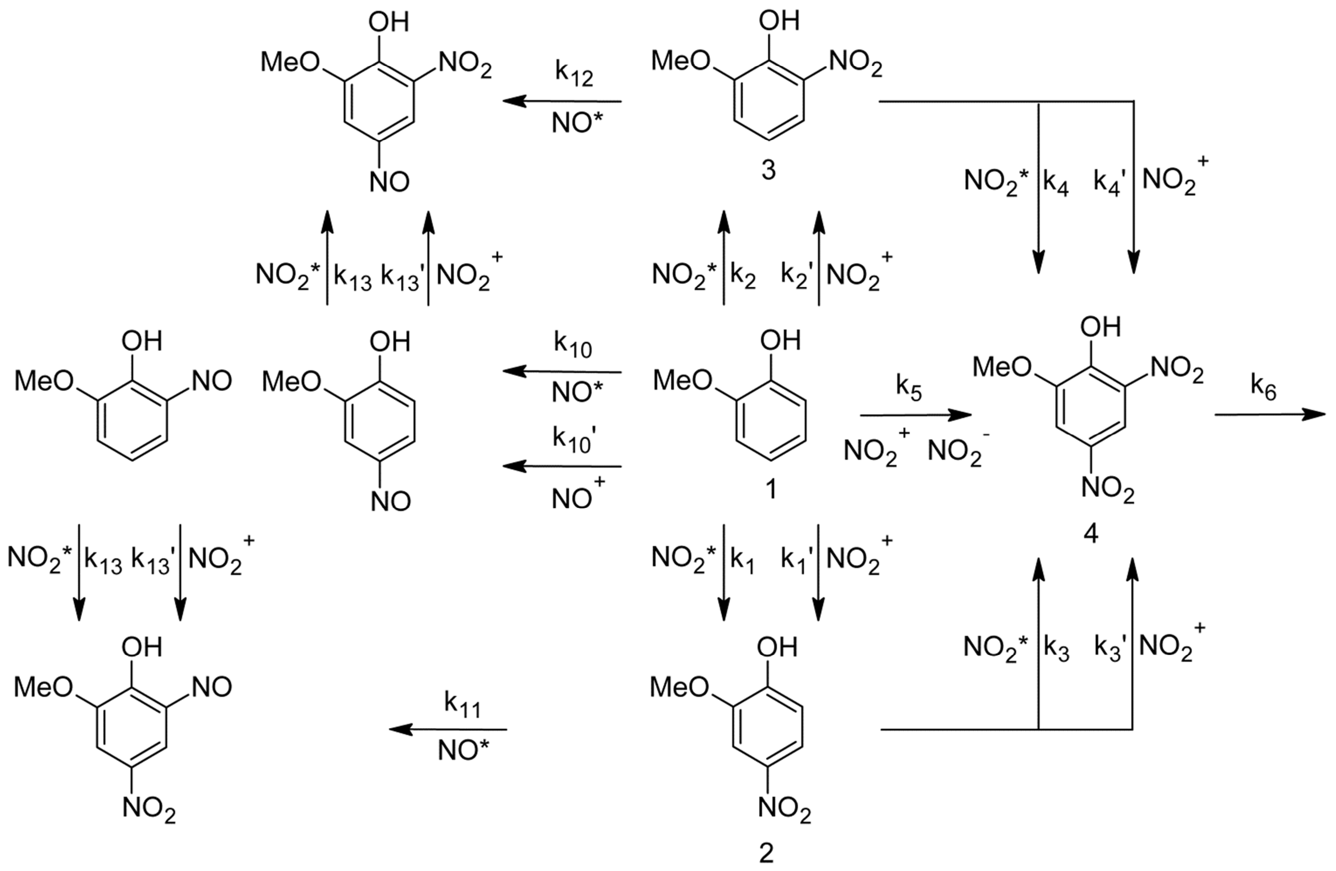

Supplementary Figure 1 | Proposed reaction mechanism of guaiacol transformations in acidic aqueous solution.

1 guaiacol (GUA), **2** 4-nitroguaiacol (4NG), **3** 6-nitroguaiacol (6NG), and **4** 4,6-dinitroguaiacol (DNG). The model does not distinguish between 4- and 6-nitrosoguaiacol (NOG).

Supplementary Table 1 | Best-fit kinetic parameters. Best-fit kinetic rate constants with 95 % confidence are valid at the experimental conditions applied, i.e. 25°C and pH 4.5.

Reaction	r_i	k_i		unit
1	$k_1 \cdot [GUA] \cdot [NO_2^{\bullet}]$	$(4.008148 \pm 0.000006) \cdot 10^9$	*	$L\ mol^{-1}\ s^{-1}$
1'	$k_1' \cdot [GUA] \cdot [NO_2^{+}]$	$(2.518 \pm 0.005) \cdot 10^5$		$L\ mol^{-1}\ s^{-1}$
2	$k_2 \cdot [GUA] \cdot [NO_2^{\bullet}]$	$(5.735602 \pm 0.000003) \cdot 10^9$	*	$L\ mol^{-1}\ s^{-1}$
2'	$k_2' \cdot [GUA] \cdot [NO_2^{+}]$	$(4.065 \pm 0.006) \cdot 10^5$		$L\ mol^{-1}\ s^{-1}$
3	$k_3 \cdot [4NG] \cdot [NO_2^{\bullet}]$	$(7.04158 \pm 0.00004) \cdot 10^8$	*	$L\ mol^{-1}\ s^{-1}$
3'	$k_3' \cdot [4NG] \cdot [NO_2^{+}]$	$(1.417 \pm 0.003) \cdot 10^1$		$L\ mol^{-1}\ s^{-1}$
4	$k_4 \cdot [6NG] \cdot [NO_2^{\bullet}]$	$(1.19040 \pm 0.00004) \cdot 10^8$	*	$L\ mol^{-1}\ s^{-1}$
4'	$k_4' \cdot [6NG] \cdot [NO_2^{+}]$	$(7.01 \pm 0.01) \cdot 10^2$		$L\ mol^{-1}\ s^{-1}$
5	$k_5 \cdot [GUA] \cdot [NO_2^{+}] \cdot [NO_2^{-}]$	$(3.034 \pm 0.006) \cdot 10^3$		$L^2\ mol^{-2}\ s^{-1}$
6	$k_6 \cdot [DNG]$	$(6.6 \pm 0.4) \cdot 10^{-6}$		s^{-1}
10	$k_{10} \cdot [GUA] \cdot [NO^{\bullet}]$	$(6.648324 \pm 0.000004) \cdot 10^9$	*	$L\ mol^{-1}\ s^{-1}$
10'	$k_{10}' \cdot [GUA] \cdot [NO^{+}]$	$(5.461 \pm 0.008) \cdot 10^2$		$L\ mol^{-1}\ s^{-1}$
11	$k_{11} \cdot [4NG] \cdot [NO^{\bullet}]$	$(9.18238 \pm 0.00004) \cdot 10^8$	*	$L\ mol^{-1}\ s^{-1}$
12	$k_{12} \cdot [6NG] \cdot [NO^{\bullet}]$	$(3.856275 \pm 0.000005) \cdot 10^9$	*	$L\ mol^{-1}\ s^{-1}$
13	$k_{13} \cdot [NOG] \cdot [NO_2^{\bullet}]$	$(1.0946999 \pm 0.0000005) \cdot 10^{10}$	*,†	$L\ mol^{-1}\ s^{-1}$
13'	$k_{13}' \cdot [NOG] \cdot [NO_2^{+}]$	$(4.09 \pm 0.01) \cdot 10^4$	†	$L\ mol^{-1}\ s^{-1}$

* Kinetic rate constants are correlated with the apparent production rate constants of $NO_2^{\bullet}$ or $NO^{\bullet}$. Still, their values are set reasonable for radical reactions, the ratios between them are reliable, and this does not affect the conclusions stated in the manuscript.

† Apparent kinetic rate constants for nitration of all NOGs (4- and 6-nitrosoguaiacol) are reported.

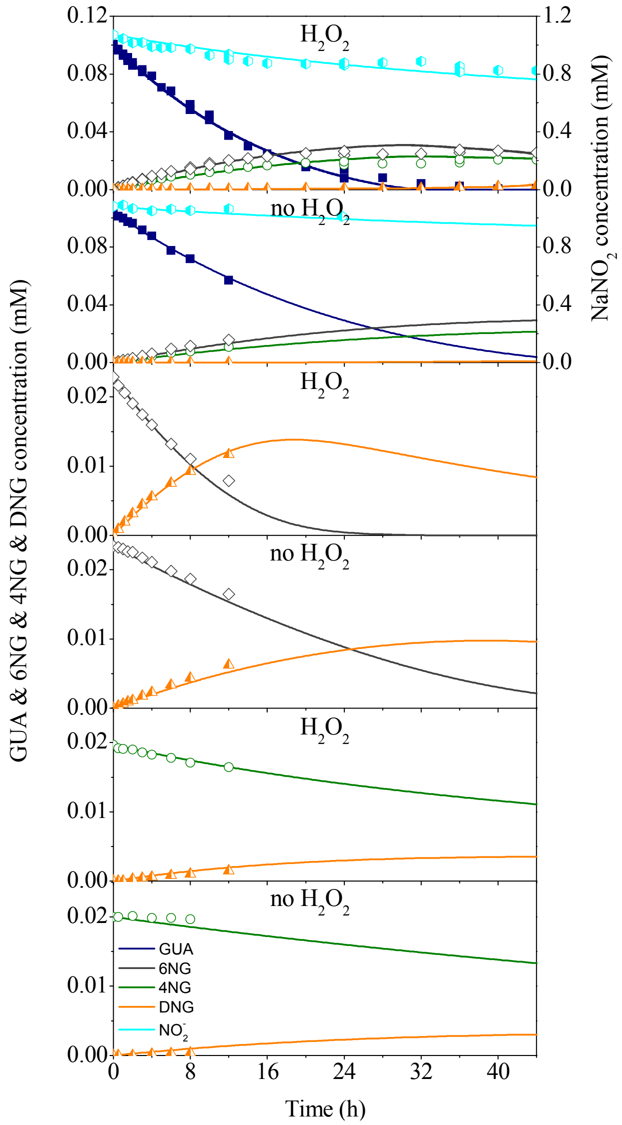

Supplementary Figure 2 | Experimental data with modelled curves in the dark. Nitration of GUA (filled dark blue squares), 6NG (open dark grey diamonds), and/or 4NG (open green circles) in acidic H_2SO_4 solution (pH 4.5) in the dark at 25 °C upon addition of 1 mM $NaNO_2$ (halved cyan hexagons) with and without 1 mM H_2O_2. DNG is represented by halved orange triangles. Characters represent experimental data points and solid lines are fits according to the proposed reaction mechanism.

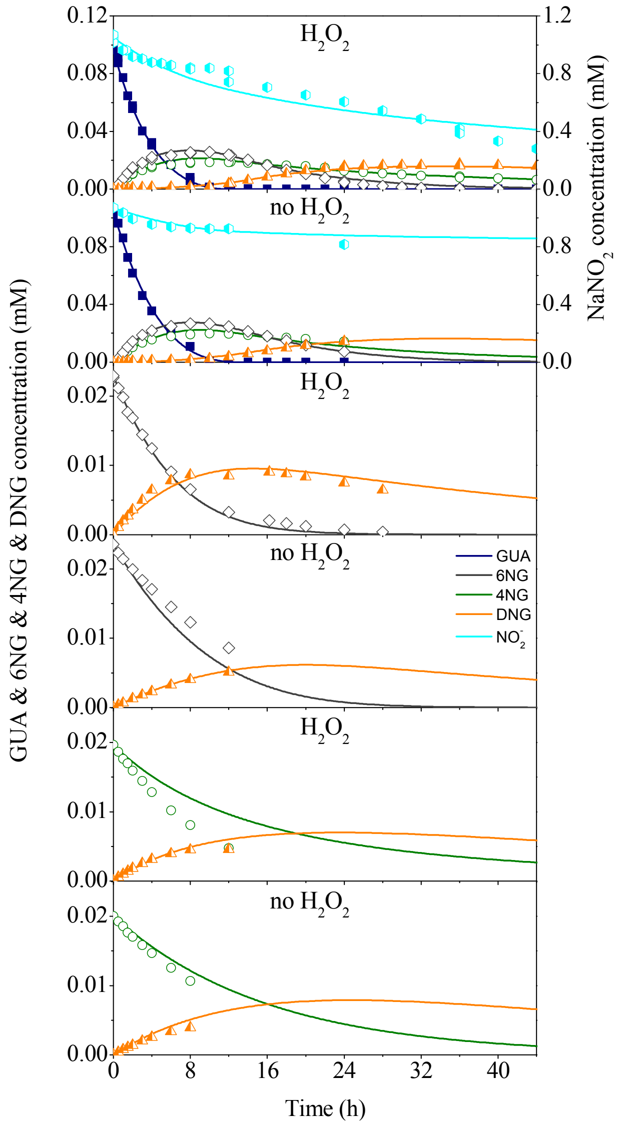

Supplementary Figure 3 | Experimental data with modelled curves under simulated sunlight conditions. Nitration of GUA (filled dark blue squares), 6NG (open dark grey diamonds), and/or 4NG (open green circles) in acidic H_2SO_4 solution (pH 4.5) under simulated sunlight conditions at 25 °C upon addition of 1 mM $NaNO_2$ (halved cyan hexagons) with and without 1 mM H_2O_2. DNG is represented by halved orange triangles. Characters represent experimental data points and solid lines are fits according to the proposed reaction mechanism.

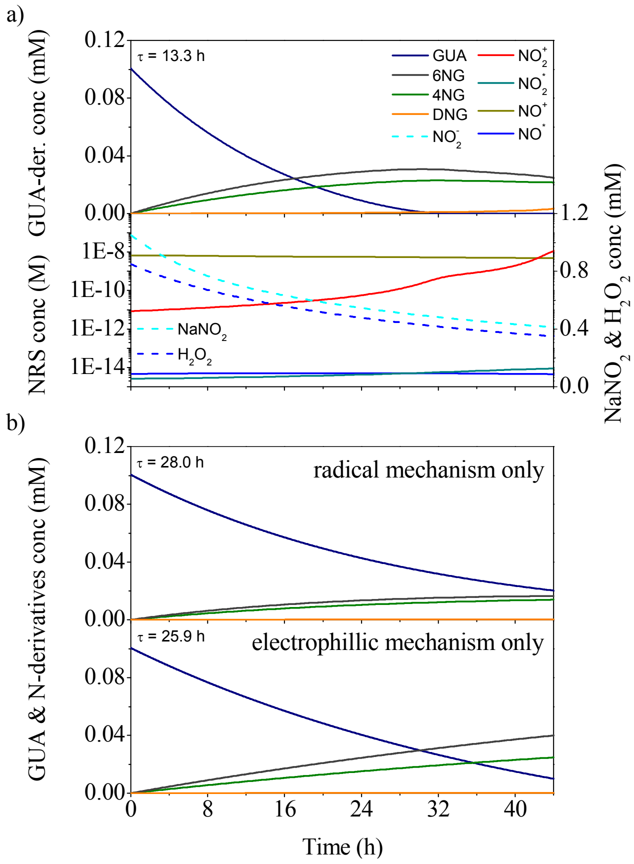

Supplementary Figure 4 | Modelled concentration profiles in the dark. a) Modelled nitration of GUA (solid blue line) in the dark under the experimental conditions upon addition of H_2O_2 (dashed blue line). b) Modelled nitration of GUA according to radical and electrophilic mechanism only. Other symbols used: 4NG (solid green line), 6NG (solid dark grey line), DNG (solid orange line), $NO_2^{\bullet}$ (solid dark cyan line), $NO^{\bullet}$ (solid blue line), NO_2^+ (solid red line), NO^+ (solid dark yellow line), and $NaNO_2$ (dashed cyan line). GUA lifetimes (τ) are also assessed.

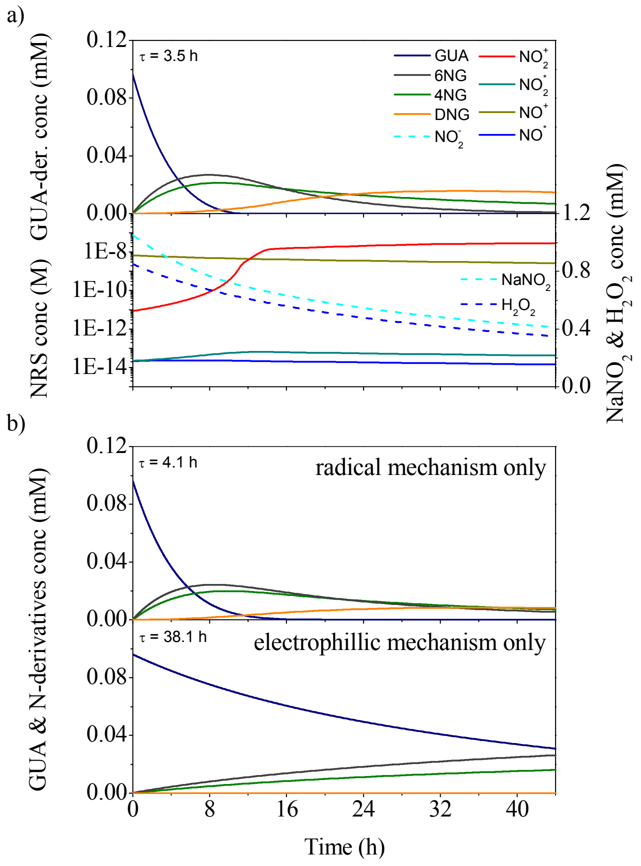

Supplementary Figure 5 | Modelled concentration profiles under simulated sunlight conditions. a) Modelled nitration of GUA (solid blue line) under illuminated experimental conditions upon addition of H_2O_2 (dashed blue line). b) Modelled nitration of GUA according to radical and electrophilic mechanism only. Other symbols used: 4NG (solid green line), 6NG (solid dark grey line), DNG (solid orange line), $NO_2^•$ (solid dark cyan line), $NO^•$ (solid blue line), NO_2^+ (solid red line), NO^+ (solid dark yellow line), and $NaNO_2$ (dashed cyan line). GUA lifetimes (τ) are also assessed.
